# Response of Pembrolizumab Alone for Non-small Cell Lung Cancer With Brain Metastasis: A Case Report and Literature Review

**DOI:** 10.3389/fonc.2020.577159

**Published:** 2020-10-26

**Authors:** Eric A. Marvin, Kimberley L. Furrow, Ayesha Kar, Joshua A. Cuoco

**Affiliations:** ^1^Carilion Clinic, Section of Neurosurgery, Roanoke, VA, United States; ^2^Virginia Tech Carilion School of Medicine, Roanoke, VA, United States; ^3^Virginia Tech School of Neuroscience, Blacksburg, VA, United States

**Keywords:** pembrolizumab, immunotherapy, non-small cell lung cancer, central nervous system metastases, brain metastases (BM)

## Abstract

Treatment of brain metastases often includes surgical resection, chemotherapeutics and radiotherapy. Given the difficulty in obtaining therapeutic levels of medications within the immune-privileged central nervous system, chemotherapy as a stand-alone treatment modality for brain metastases is an uncommon option. However, there is a growing body of evidence to suggest that immunomodulatory agents can induce a robust immune response in the central nervous system. Here, we describe a 68-year old male who presented with radiographic evidence of new and enlarging lung nodules with mediastinal adenopathy. Lung biopsy was consistent with adenocarcinoma. Immunohistochemical staining demonstrated high expression of programmed cell death protein 1 with a tumor proportion score of 100%. Surveillance magnetic resonance imaging of the brain demonstrated a single enhancing 11 × 7 × 12 mm lesion along the mesial surface of the right frontal lobe. The patient deferred surgical resection as well as stereotactic radiosurgery but agreed to treatment with pembrolizumab. Repeat magnetic resonance imaging at 3-months after initiation of treatment demonstrated complete radiographic resolution of the brain lesion. To our knowledge, this is one of only a few reports in the current literature to document complete resolution of non-small cell lung cancer brain metastasis with pembrolizumab alone. We discuss the emerging literature regarding the efficacy of pembrolizumab in the treatment of brain metastases, central nervous system penetration, and emerging new treatment paradigms involving novel immunotherapy agents.

## Background

Brain metastases are a common finding in non-small cell lung cancer (NSCLC) occurring in ~16–44% of patients (1–4). Improvement in systemic disease control and widespread availability of imaging has likely contributed to the increasing incidence of brain metastases (5, 6). While the advancement of immunotherapy has begun to become an integral part of the treatment paradigm for NSCLC, brain metastases continues to be managed with surgical resection and radiotherapy (7). Treatment of a single metastatic intracranial lesion often involves surgical resection in the appropriate patient population with accessible lesions followed by stereotactic radiosurgery (SRS). Historically, whole brain radiation therapy (WBRT) was the standard treatment of choice for the management of metastatic disease dissemination of the brain parenchyma with the aim in managing both the visible metastases as well as disease at the cellular level (8). However, due to the consequential cognitive decline experienced by many patients with WBRT, the field has moved away from WBRT and toward local control with SRS (9, 10). In patients deemed poor surgical candidates or lesions that are either deep seated or in eloquent areas, SRS alone has become a reasonable and reliable treatment option replacing WBRT in many patient subsets (8, 11).

While chemotherapy or immunotherapy are effective and necessary in the treatment and control of primary NSCLC, the literature depicting pembrolizumab as stand-alone treatment modality for NSCLC brain metastases is sparse (12–17). Limited studies have demonstrated control of brain metastases when used with WBRT and after failure of other treatment regimens (12, 13). As the use of immune checkpoint inhibitors have become more widespread, research has demonstrated some activity against brain metastases; however, given the lack of response by many patients, the effectiveness of immunotherapy in the treatment of intracranial metastatic disease has yet to be fully elucidated (14–17). Here, we describe a patient with newly diagnosed NSCLC who desired to follow his brain metastasis with serial imaging after initiating pembrolizumab immunotherapy. Imaging to date has demonstrated a complete radiographic response. To our knowledge, this is one of only a few reports in the current literature to document complete resolution of NSCLC brain metastasis with pembrolizumab alone.

## Case Presentation

**Figure 1:**
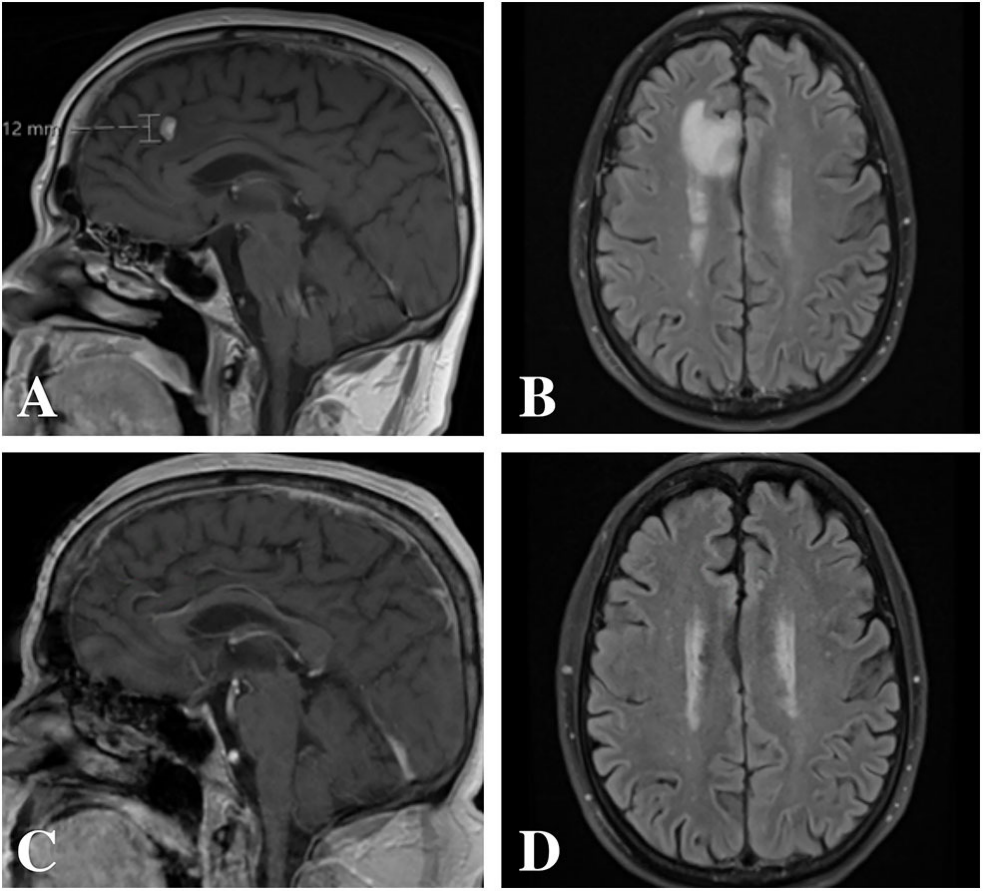
Magnetic resonance imaging pre- and post-treatment. **(A,B)** Pre-treatment imaging demonstrated an avidly enhancing 11 × 7 × 12 mm lesion along the mesial surface of the right frontal lobe within the cingulate sulcus with surrounding vasogenic edema. **(C,D)** Post-treatment imaging with complete radiographic resolution of the lesion and associated vasogenic edema.

A 68-year old male, with a past medical history remarkable for an 88 pack-year smoking history and chronic obstructive pulmonary disease on supplemental oxygen, underwent an annual lung cancer screening with a computed tomography (CT) scan of the chest ordered by his primary care physician. CT demonstrated an increase in previously identified lung nodules as well as five new nodules, fluid within the right lung and ipsilateral mediastinal adenopathy. Subsequent bronchoscopy and lymph node biopsies demonstrated pulmonary adenocarcinoma that was EGFR negative, ALK negative, ROS1 negative, BRAF wild type, and highly positive for programmed cell death protein 1 (PD-1) (>50%) with a tumor proportion score of 100%. Surveillance magnetic resonance imaging (MRI) of the brain (1 mm cuts) demonstrated a single, avidly enhancing 11 × 7 × 12 mm lesion along the mesial surface of the right frontal lobe within the cingulate sulcus with surrounding vasogenic edema (Figures 1A,B). As such, his primary disease was considered stage IV (T0N2M1b). He was started on pembrolizumab immunotherapy by medical oncology and referred to neurosurgery for further treatment considerations of his single metastatic intracranial lesion.

Risks and benefits of treating and withholding treatment for the single metastatic intracranial lesion were discussed at length with the patient. Given the patient's poor pulmonary status and the location of the brain metastasis, we recommended SRS. However, the patient refused treatment other than pembrolizumab but was amenable to considering intervention should the lesion progress on follow-up imaging. Four cycles of pembrolizumab (200 mg every 3 weeks) were planned with follow up MRI thereafter to revisit treatment options based upon imaging findings. Repeat MRI (1 mm cuts) 3 months after initiating pembrolizumab demonstrated complete radiographic resolution of the intracranial lesion and associated vasogenic edema without any new interim findings (Figures 1C,D). Follow-up positron-emission tomography 9 months after starting treatment demonstrated no focal accumulations within the brain parenchyma but with persistent yet decreased uptake within the right upper lobe, two areas of new uptake and interval resolution of mediastinal and hilar lymph node activity. The patient completed ten cycles of pembrolizumab without any reported clinical sequelae throughout his treatment course. At 1-year follow up after initial diagnosis, the patient was doing well with his pulmonary function at baseline.

## Discussion

Metastatic brain tumors continue to be the leading cause of central nervous system malignancy in adults with NSCLC accounting for more than 30–60% of all cases (18). The development of immunotherapies, specifically those targeted at immune checkpoints, has led to significant progress in the field of medical oncology, especially for patients with advanced NSCLC. However, there is limited data on the permeability of pembrolizumab and other immune checkpoint inhibitors at the blood-brain barrier. Traditional chemotherapy agents, including platinum analogs, paclitaxel, docetaxel, gemcitabine and other anti-cancer drugs used to treat adenocarcinoma, have poor penetration and duration into brain tumor tissues at sufficient concentrations to cause significant anti-tumor effects (19–23). Immunotherapy for the treatment of NSCLC, including agents such as pembrolizumab, nivolumab, atezolizumab, and durvalumab, are fairly novel agents used in the treatment of metastatic NSCLC. Central nervous system penetration and efficacy have yet to been substantiated, though there are reports that suggest potential activity and therapeutic responses (14–16).

Pembrolizumab is a highly selective humanized monoclonal IgG4-kappa isotope antibody that is directed against PD-1 (24). PD-1 is located on T lymphocytes and pro-B lymphocytes and interacts with two ligands: programmed cell death ligand 1 (PD-L1) and ligand 2. T-cell function becomes inhibited when PD-L1 binds PD-1 (25). As such, pembrolizumab inhibits formation of the PD-1: PD-L1 complex, which allows for an improved T-cell response to tumor cells (25). As the KEYNOTE-001 clinical trial demonstrated, 23.2% of NSCLC patients not treated previously with chemotherapy and 15.5% of previously treated patients were alive after 5 years with pembrolizumab monotherapy (7). This is a significant improvement to a historical 5-year survival of 5.5% prior to immunotherapy (7). However, the question as to whether immunotherapy can be a stand-alone treatment for single brain metastases with the goal of avoiding invasive surgical procedures and radiation has yet to be answered. There are few case reports and one prospective trial of complete resolution of metastatic brain lesions in NSCLC, though many of these patients also received some type of additional therapy including WBRT, SRS or surgical resection (14–16, 22, 26, 27). Research is sparse in detailing the effectiveness of immunomodulatory agents in treatment of NSCLC brain metastases in the absence of previous radiation, though recent reports have begun to highlight this area (1, 14–16).

Pembrolizumab may be beneficial for stage IV NSCLC with brain metastasis as a monotherapy without the use of radiotherapy. To date, there is only one prospective study by Goldberg et al. investigating the efficacy of pembrolizumab in patients with NSCLC or melanoma with untreated brain metastases measuring 5–20 mm (15). The study consisted of two cohorts: cohort one was for patients with PD-L1 expression of at least 1% and cohort two was for patients with PD-L1 <1%. Eleven of 37 patients (29.7%) in cohort one exhibited a brain metastasis response on subsequent radiographic imaging. Specifically, seven patients exhibited a partial response on subsequent imaging whereas four patients exhibited complete radiographic resolution at a median follow up time of 1.8 months after the initiation of treatment. Moreover, none of the five patients in cohort two demonstrated a response. Interestingly, median overall survival did not differ significantly between patients with PD-L1 expression of at least 1% vs. PD-L1 expression <1% (15).

The resolution of intracranial lesions on follow-up imaging suggests permeability of the blood-brain barrier to pembrolizumab, though numerous questions remain to be answered regarding which patients may have a propensity to respond, and which imaging, clinical findings, or biomarkers may correlate with central nervous system efficacy (1). Accurate prediction of which patients with NSCLC brain metastases will respond to immunotherapy may help prevent or at least delay unnecessary invasive therapies. While some studies suggest that NSCLC patients with high tumor PD-1 expression show better response rates and longer survival with immunotherapy, this does not necessarily translate to the response rate of NSCLC brain metastases (28). The heterogeneity of PD-1 receptor status of tumor cells as well as potential variations in the immunophenotypes of the receptors between the primary lesion compared to the metastatic lesions make generalizations difficult (1, 29). Though receptor status of the brain metastases is inevitably of interest, other research has focused on the PD-1 status of the tumor infiltrating lymphocytes (TIL) in metastatic brain lesions. Higher TIL scores in metastatic brain lesions have correlated to improved progression free survival; however, other studies indicate that TIL scores of the metastatic brain lesion may not correlate with the primary lesion (17, 30). Moreover, the variability of currently available assays makes definitive analysis, and, thus, clinical correlations difficult (31).

## Conclusions

As the median survival of NSCLC continues to improve with increasingly efficacious therapies, addressing secondary anticipated effects of longer survival times such as an increasing rate of brain metastases will become a more demanding issue. Patients choosing immunotherapy and forgoing more invasive modalities, such as surgical resection or radiation treatment, for asymptomatic, incidental lesions may become a challenge that needs further investigation and substantiation. Delaying surgical or radiation treatment with the hope that immunotherapy will successfully treat the metastatic process is only a feasible concept if research can accurately identify likely responders. A non-aggressive approach toward an aggressive lesion may lead to increased growth of the lesion and associated vasogenic edema that is often confounded by neurologic symptomatology, seizures, cognitive decline, and deterioration in the patient's performance status (32). Certainly, there is a direct correlation between performance status and clinical outcomes (33). Indeed, an improved understanding of immunotherapy and its effectiveness in the central nervous system may lead to better treatment paradigms for metastatic brain cancer and, ultimately, improved clinical outcomes.

## Data Availability Statement

The original contributions presented in the study are included in the article/supplementary material, further inquiries can be directed to the corresponding author/s.

## Ethics Statement

The patient described in this report provided written informed consent for its publication.

## Author Contributions

EM, KF, AK, and JC: provided substantial contributions to the conception and design of the manuscript, contributed to manuscript revision, read, and approved the submitted version, agree to be accountable for all aspects of the work ensuring that questions related to the accuracy or integrity of any part of the work are investigated and resolved. All authors contributed to the article and approved the submitted version.

## Conflict of Interest

The authors declare that the research was conducted in the absence of any commercial or financial relationships that could be construed as a potential conflict of interest.

## Abbreviations

NSCLC: non-small cell lung cancer
SRS: stereotactic radiosurgery
WBRT: whole brain radiation therapy
CT: computed tomography
MRI: magnetic resonance imaging
PD-1: programmed cell death protein 1
PD-L1: programmed cell death ligand 1
TIL: tumor infiltrating lymphocytes.
